# Are Utilitarian/Deontological Preferences Unidimensional?

**DOI:** 10.3389/fpsyg.2016.01228

**Published:** 2016-08-17

**Authors:** Michael Laakasuo, Jukka Sundvall

**Affiliations:** Cognitive Science Unit, University of HelsinkiHelsinki, Finland

**Keywords:** moral psychology, psychometrics, utilitarianism, deontology, factor-analysis

## Abstract

Utilitarian versus deontological inclinations have been studied extensively in the field of moral psychology. However, the field has been lacking a thorough psychometric evaluation of the most commonly used measures. In this paper, we examine the factorial structure of an often used set of 12 moral dilemmas purportedly measuring utilitarian/deontological moral inclinations. We ran three different studies (and a pilot) to investigate the issue. In Study 1, we used standard Exploratory Factor Analysis and Schmid-Leimann (*g* factor) analysis; results of which informed the *a priori* single-factor model for our second study. Results of Confirmatory Factor Analysis in Study 2 were replicated in Study 3. Finally, we ran a weak invariance analysis between the models of Study 2 and 3, concluding that there is no significant difference between factor loading in these studies. We find reason to support a single-factor model of utilitarian/deontological inclinations. In addition, certain dilemmas have consistent error covariance, suggesting that this should be taken into consideration in future studies. In conclusion, three studies, pilot and an invariance analysis, systematically suggest the following. (1) No item needs to be dropped from the scale. (2) There is a unidimensional structure for utilitarian/deontological preferences behind the most often used dilemmas in moral psychology, suggesting a single latent cognitive mechanism. (3) The most common set of dilemmas in moral psychology can be successfully used as a unidimensional measure of utilitarian/deontological moral inclinations, but would benefit from using weighted averages over simple averages. (4) Consideration should be given to dilemmas describing infants.

## Introduction

Utilitarianism is an ethical philosophy stating that aggregate welfare or “good” should be maximized and that suffering or “bad” should be minimized. It is usually contrasted with deontological philosophy, which states that there are inviolable moral rules that do not change depending on the situation (Greene, 2007b). From a utilitarian perspective, murder can be justified if its benefits outweigh the costs, for instance, if a murder of a dangerous criminal saves lives. From a deontological perspective, an act is simply right or wrong despite its consequences. Deontologists argue that if a moral rule can be violated in one situation, it can be violated in any situation, and therefore stops being a moral rule. For example, “do not kill” is a classic absolute deontological rule, and thus murder is always wrong from a deontological perspective even if it saves lives. For a utilitarian, the ends justify the means whereas for a deontologist they do not.

In recent years, these two moral preferences have been studied in the field of moral psychology by using vignettes, stories, and dilemmas (e.g., Bartels and Pizarro, 2011; Djeriouat and Trémolière, 2014; Lee and Gino, 2015; see Christensen and Gomila, 2012 for a review). The type of stimulus material most often used are sets of dilemmas that pit utilitarian and deontological inclinations against each other in an emotionally engaging way. These dilemmas often describe a situation where the moral agent (or the participant) has the option to kill an innocent person with her/his own actions, in order to save the lives of several others. Such dilemmas, with a clear utilitarian motivation to harm, have been called *high-conflict dilemmas* because they require an emotionally taxing personal engagement. That is to say, they create a conflict between utilitarian and deontological inclinations by juxtaposing them on the same continuum (e.g., Greene et al., 2008; Lee and Gino, 2015). In contrast, dilemmas where the harm is impersonal, indirect (caused by proxy), or has no utilitarian motivation, have been called low-conflict as they do not require the same type of personal engagement.

Nonetheless, reactions to high-conflict dilemmas are theoretically more interesting, as they are much more varied than reactions to low-conflict dilemmas (Greene, 2007b), and can be used to measure the strengths of the two moral inclinations or poles (Cushman and Greene, 2012). Indeed, the most commonly used set of moral dilemmas are high-conflict moral dilemmas. Furthermore, the high-conflict moral dilemmas are arguably indicating that there could be a unitary cognitive resource (“*g* factor”) latent behind both types of moral-cognitive preferences. A classic example of a high-conflict dilemma is the *footbridge dilemma*, where a runaway trolley is about to run over and kill five people. The participant has the option to push an innocent bystander down from a footbridge in front of the trolley, killing the bystander, but saving five other people from certain death. The participant is commonly asked if pushing the bystander to their death is acceptable; accepting the “sacrifice” is considered a utilitarian response. If the participant concludes that this action is not permissible, their judgment is considered to be deontological. A single scale can be used to describe the level of utilitarianism versus deontology in these responses where, as in the case of the runaway trolley, the preferences are mutually exclusive.

Utilitarian preferences have been positively linked to all three Dark Triad measures (Psychopathy, Narcissism and Machiavellianism; Bartels and Pizarro, 2011) and negatively with Honesty–Humility and harm/care ethics (Djeriouat and Trémolière, 2014). Neuroscientific research has also found that people with specific prefrontal cortex lesions prefer the utilitarian options in these dilemmas (Koenigs et al., 2007); especially if the limbic areas are unable to provide “emotional” information for the “rational” prefrontal areas. In agreement with the above, people with greater working memory capacity have been found to be more utilitarian (Moore et al., 2008). In general, it has been claimed that deontological responses to the aforementioned dilemmas are an instinctive, emotionally based “gut reaction,” and that utilitarian responses take more thought, being the more rational or non-biased decision (e.g., Greene, 2007a). However, Bauman et al. (2014) have raised concerns over the external validity of moral dilemmas as a tool for measuring moral judgments. Bauman et al. (2014) state that certain moral dilemmas are more fit for philosophical discussion than actual measurement, as the dilemmas do not resemble everyday life in a meaningful way. On the other hand, Cushman and Greene (2012) have argued that the clear–cut – and thus somewhat unrealistic – nature of moral dilemmas is exactly the reason that sheds more light on the underlying structure behind moral thought. For a clear measurement of utilitarian versus deontological tendencies, it may be that highly hypothetical scenarios are needed to get clear signals regarding the structure of our moral cognition.

Notwithstanding, up to date, there has been no standard way to pose the dilemmas or to measure utilitarian or deontological preferences. Preferences have been measured using dichotomous measures (most common) or Likert scales (See Christensen and Gomila, 2012 for review). Also, there is not a single standard set of dilemmas yet; at least three different sets of dilemmas have been used in previous studies (e.g., Greene et al., 2008; Bartels and Pizarro, 2011 and Lee and Gino, 2015), while these sets are partially overlapping. However, as far as we are aware, there has not been an *extensive* psychometric assessment on validity of the scales as psychometric instruments so far. Given the aforementioned associations between moral inclinations, emotion, memory, personality, and brain lesions, it becomes increasingly important to have validated instruments for measurement. Though the dilemmas share a common form, they describe a variety of different situations and thus have qualitative differences. It is possible that some items are better at measuring the moral inclinations than others. Furthermore, some of them may form separate sub-factors relevant for moral thought. In the following three studies, we used a set of 12 high-conflict dilemmas developed by Greene et al. (2008) to measure moral inclinations and examine the factorial structure of responses to these dilemmas. We chose this set as it has been used in several other studies and utilizes the more interesting dilemma type. We started with the standard assumption made in the literature, that these dilemmas are part of a single-factor measurement model; i.e., they measure utilitarian versus deontological inclinations in a uniform way. Different alternatives of this assumption are further explored.

## Pilot Study/Instrument Calibration

We collected a small sample to test if our initial assumption regarding the unidimensional structure behind the dilemmas would be supported. We further aimed to make sure that our analyses would have an empirical foundation.

### Ethical Statement

All local laws regarding ethics for social science research were followed in full. All participation was fully voluntary and participants were informed about their right to opt out at any point without penalties. All materials and a study protocol were reviewed and approved by the University of Helsinki Ethical Review Board in Humanities and Social and Behavioral sciences.

### Methods

#### Participants and Procedure

Fifty-seven Finnish people were recruited to an anonymous Internet study on Facebook. No identifying information, not even gender, was collected. After providing their informed consent, participants read the instructions and responded to the 12 moral dilemmas using a 7 point Likert scale (Greene et al., 2008).

#### Materials

##### Moral preference measure

We used 12 high-conflict moral dilemmas adopted from Greene et al. (2008). The dilemmas are presented in Supplementary Material. In each of the dilemmas, the participant was instructed to assume the role of the moral agent in the scenario. The moral dilemmas deal with different topics from military emergencies to trekking accidents and even situations where the agent has to consider sacrificing their own child. Each of the dilemmas described a morally ambiguous situation where the moral agent has to judge how acceptable it is to kill or injure one person in order to save multiple others (or to prevent a person from suffering before inevitable death). The utilitarian option in each dilemma has the moral agent carry the harm out with their own hands – e.g., pushing a person off a footbridge in front of a trolley.

All questions were framed in the following manner: “How acceptable is it for you to do X [e.g., ‘push the bystander off the footbdrige’]?” All questions were anchored from 1: ‘not at all acceptable’ to 7: ‘totally acceptable.’ By conventional standards the sacrificial dilemmas had a good inter-item reliability (Cronbach’s α = 0.92). Since Cronbach’s α is known to have psychometric problems (e.g., Zinbarg et al., 2005; Dunn et al., 2014), we also calculated Tarkkonen’s ρ for the items and their internal reliability (ρ = 0.87), which indicated acceptable internal consistency as well.

### Results of Pilot Study

Theoretically, we assumed that there would only be one factor for the dilemmas which was also suggested by Tarkkonen’s ρ. We ran a parallel analysis to confirm this. Based on the Eigenvalue criterion, the recommendation was one factor and based on Optimal Coordinates, Acceleration Factor and Parallel analysis the recommendation was also a single-factor solution. We also ran a Maximum Likelihood (ML) estimated exploratory factor analysis with VARIMAX rotation on our pilot data. According to the analysis, all items loaded on a single factor (all loadings >0.55) with an eigenvalue of 6.6, while the next possible extracted factor had an eigenvalue of 0.85. We concluded that the small sample pilot data suggests that it is worth pursuing a single-factor solution for the factorial structure of the 12 most common dilemmas in the field of Moral Psychology. For an example of a graphical presentation for Parallel Analysis see the results of Study 1.

## Study 1

Since our pilot study was an *ad hoc* online study, we decided to collect a larger laboratory data in conjunction with other experiments (reported elsewhere), assuming that laboratory data would be less noisy and a more uniformly controlled environment than what, we would have in online questionnaires.

### Method

#### Participants and Procedure

Participants were recruited through the social media and from public libraries in the city center of Helsinki. One hundred and fifty-six (*N* = 156; 65 male; *M*_age_ = 26.83; *SD*_age_ = 8.81; *range* = 18 – 62) people participated in laboratory experiments, where they filled in the moral dilemmas in conjunction with other tasks unrelated to the aims of this study (i.e., evaluating emotions of faces and other emotional intelligence tasks). All dilemmas were presented in a fully randomized order. Participants answered the dilemmas in their native language and were compensated an average of 2.5€ for their time.

#### Materials

##### Moral preference measure

We used the same materials as in the Pilot Study above. For a full description of the materials, see Pilot Study and Supplementary Material. By conventional standards, the high-conflict dilemmas had a good inter-item reliability (Cronbach’s α = 0.89). We also calculated Tarkkonen’s ρ (ρ = 0.82), which indicated acceptable internal consistency as well.

### Results

**FIGURE 1:**
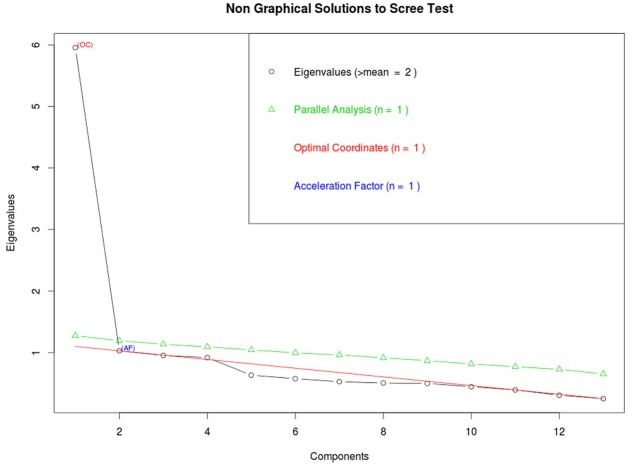
**Parallel analysis results for number of factors for the data presented in Study 1**.

Theoretically, we assumed that there would only be one factor, as also suggested by Tarkkonen’s ρ. Additionally, we ran a parallel analysis to confirm this result. Based on Eigenvalue criterion, the recommendation was two factors and based on Optimal Coordinates, Acceleration Factor and Parallel analysis, one factor (See **Figure 1**).

Next, we ran a ML estimated exploratory factor analysis for a single-factor solution. All items loaded on the factor with decent values (0.45 – 0.74). We also ran the two- and three-factor analyses with no rotation, varimax and promax rotations (for full factor loadings, see **Tables 1** and **2**).

**Table 1:** Solutions of exploratory unidimensional and two-dimensional factor analyses.

			Two-dimensional solutions						
				
	Unidimensional solution		Unrotated		Varimax		Promax		
					
Item	Loading	error	F1	F2	F1	F2	F1	F2	η^2^
Crying baby	0.66	0.56	**0.72**	-0.37	0.28	**0.76**		**0.82**	0.65
Euthanasia	0.65	0.58	**0.63**		**0.53**	0.35	**0.51**	0.16	0.40
Foot bridge	0.50	0.75	**0.51**		0.31	0.42		0.37	0.27
Lawrence of Arabia	0.69	0.53	**0.68**	0.35	**0.74**	0.20	**0.86**		0.58
Terrorist’s son	0.51	0.74	**0.50**		**0.47**	0.23	**0.50**		0.27
Life boat	0.75	0.43	**0.73**		**0.64**	0.38	**0.65**		0.55
Safari	0.72	0.48	**0.71**		**0.61**	0.40	**0.59**		0.52
Sacrifice	0.72	0.48	**0.78**	-0.35	0.35	**0.78**		**0.82**	0.74
Sophie’s choice	0.48	0.77	**0.52**	-0.40		**0.65**		**0.77**	0.43
Submarine	0.79	0.38	**0.77**	0.22	**0.71**	0.36	**0.75**		0.64
Vaccine	0.69	0.52	**0.68**	0.35	**0.74**	0.20	**0.86**		0.59
Vitamins	0.46	0.78	**0.45**		0.37	0.27	0.34		0.21


*Loadings <|0.20| not printed, loadings >|0.45| have been highlighted.*

**Table 2:** Solutions of exploratory three-dimensional factor analyses.

	Three-dimensional solutions									
		
	Unrotated			Varimax			Promax			
				
Item	F1	F2	F3	F1	F2	F3	F1	F2	F3	η^2^
Crying baby	**0.71**	-0.38		0.33	**0.72**			**0.75**		0.65
Euthanasia	**0.66**		-0.39	**0.73**	0.27		**0.76**			0.60
Foot bridge	**0.51**		0.29		0.42	0.42		0.35	0.43	0.36
Lawrence of Arabia	**0.68**	0.31		**0.49**		**0.58**	0.31		**0.64**	0.59
Terrorist’s son	**0.50**		0.42		0.22	**0.61**			0.72	0.44
Life boat	**0.75**		-0.26	**0.74**	0.29	0.21	**0.71**	0.08		0.67
Safari	**0.71**			**0.45**	0.36	0.44	0.26	0.16	0.42	0.52
Sacrifice	**0.77**	-0.39		0.29	**0.77**	0.28		**0.77**		0.75
Sophie’s choice	**0.51**	-0.40		0.22	**0.62**	0.01		**0.70**		0.43
Submarine	**0.78**	0.22		**0.70**	0.28	0.34	**0.61**		0.27	0.68
Vaccine	**0.68**	0.31	0.20	0.48		0.59	0.30		**0.65**	0.60
Vitamins	**0.46**		0.28		0.27	0.44			**0.49**	0.29


*Loadings smaller than |0.2| are not printed, Loadings ≥0.45 are highlighted.*

The results of the unrotated two- and three-factor solutions indicate that there would only be one factor on which all the items load with decent values (from 0.45 to 0.77), whereas all the other loadings on the other factors would be relatively weak (<|0.42|). Varimax and promax rotations suggest that the second factor would consist of four items: Crying Baby, Footbridge, Sacrifice, and Sophie’s Choice. Three out of these four items deal with situations where the participant has to think about sacrificing children (own or not). However, since the footbridge dilemma has nothing to do with children, there is no theoretical reason to separate these four items from the other eight.

The Varimax-rotated three-dimensional solution was the most ambiguous theoretically. This solution suggests that the Euthanasia, Life Boat, Safari and Submarine dilemmas would form one factor, and the four dilemmas that were extracted in the previous solution (Crying Baby, Footbridge, Sacrifice, and Sophie’s Choice) would consist of one factor, with all the rest (Lawrence of Arabia, Terrorist’s Son, Vaccine, and Vitamins) forming the third factor. We could not find any substantially meaningful interpretation for this solution, since it also had two relatively strong cross loadings. More specifically, the Footbridge dilemma was as much associated with child sacrifice dilemmas as with the factor consisting of Lawrence of Arabia, Terrorist’s Son, Vaccine, and Vitamins; see also loadings for the Safari dilemma.

The Promax-rotated three-factor solution was more meaningful, since it suggested that the three dilemmas involving child sacrifice (Crying Baby, Sacrifice, and Sophie’s Choice) formed a single factor, and the two dilemmas embedded in military context (Euthanasia and Submarine) would form another factor, with all the other dilemmas forming a third factor.

Finally, we ran a Schmid-Leiman factor analysis with promax rotation assuming that there would be a general factor behind the suggested two- and three-factor models to investigate; if any of the items have a stronger loading on the sub-factors rather than on the general Utilitarianism factor. In our analysis, we found that for both the two- and three-factor solutions, the *g* factor had the largest Eigenvalue (4.15 and 4.08, respectively). Only Factor 1 in the two-factor solution had an Eigenvalue slightly bigger than 1, indicating a marginal possibility for an independent factor. However, since all the items in this factor have stronger loadings on the *g* factor, there is no reason to separate factors from one another. All the other factors had Eigenvalues smaller than 1, indicating that these sub-factors contain less information than the original items, and thus support the single-factor solution (see **Table 3**). Furthermore, none of the items loaded more on the sub-factors than on the general factor.

**Table 3:** Schmid-Leiman factor analysis for two and three factors with promax rotation.

	Analysis								
	
	Two-factor solution				Three-factor solution				
		
Item	g	F1	F2	η^2^	g	F1	F2	F3	η^2^
Crying baby	**0.67**		0.45	0.65	**0.63**			0.49	0.65
Euthanasia	**0.56**	0.28		0.40	**0.56**	0.53			0.60
Foot bridge	**0.47**			0.27	**0.47**		0.28	0.23	0.36
Lawrence of Arabia	**0.59**	0.47		0.58	**0.61**	0.22	0.41		0.59
Terrorist’s son	**0.45**	0.27		0.27	**0.47**		0.46		0.44
Life boat	**0.65**	0.35		0.55	**0.65**	0.49			0.67
Safari	**0.64**	0.32		0.52	**0.64**		0.27		0.52
Sacrifice	**0.73**		0.45	0.74	**0.70**			0.50	0.75
Sophie’s choice	**0.49**		0.42	0.43	**0.46**			0.46	0.43
Submarine	**0.68**	0.41		0.64	**0.68**	0.42			0.68
Vaccine	**0.60**	0.47		0.59	**0.61**	0.20	0.42		0.60
Vitamins	**0.41**			0.21	**0.42**		0.31		0.29
Eigenvalues	4.15	1.05	0.66		4.08	0.85	0.86	0.80	


*Loadings smaller than 0.2 are not printed. Greatest loadings per item are highlighted.*

### Discussion of Results for Study 1

The results of Study 1 provide moderate support for a single-factor solution for the 12 high-conflict dilemmas. Especially the parallel analysis, together with the unrotated two- and three-factor solutions and the Schmid-Leiman factor analysis seem to support the single-factor solution. The results also seem to imply that no items should be dropped or left out of the measuring model. There is a small possibility that emotional intelligence tasks, could prime participants to give more deontological responses for the dilemmas. However, since exploratory factor analytic techniques commonly ignore the intercepts of the items, this issue is not likely to cause considerable bias in the analysis. Furthermore, since all the items were presented to the participants in randomized order, the possible priming effects are diffused equally over all of the items.

Taken together, the results of the first study give moderate support for the contention that there might be a unitary cognitive mechanism associated with the way people respond to these moral dilemmas. We also found that the three items dealing with child sacrifice (see Crying Baby, Sacrifice, and Sophie’s Choice in Supplementary Material), seemed to be more or less systematically associated with one another. We took this into account in our follow-up studies. Since an analysis based on a single laboratory data set offers only limited support for any conclusions, we collected another data to investigate if the results would replicate in a confirmatory factor analysis (CFA).

## Study 2

**FIGURE 2:**
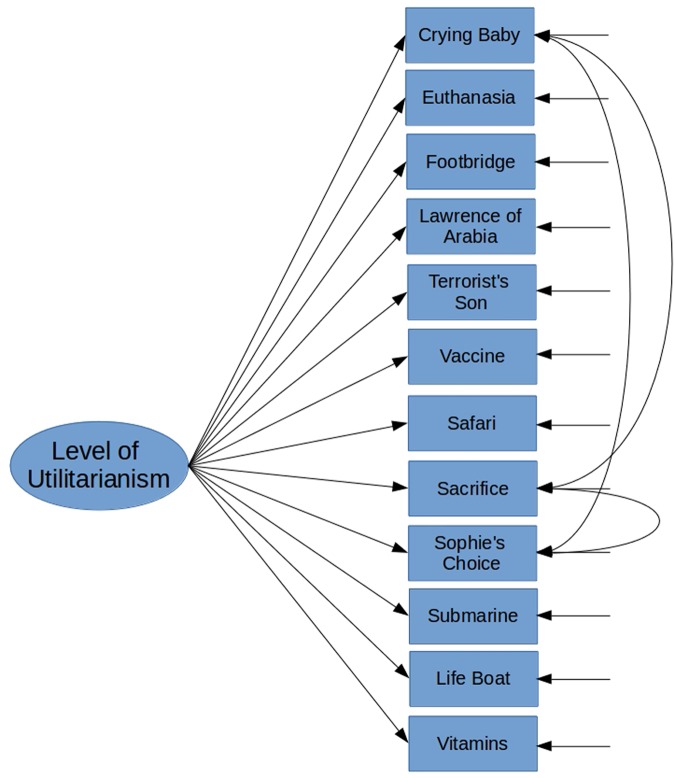
***A priori* model for confirmatory factor analysis for Study 2, based on the results of Study 1**.

The results of Study 1 imply partly that there is a unitary single-factor structure behind the items measuring utilitarianism in sacrificial moral dilemmas. We therefore decided to run a CFA hypothesizing that all items would load on a single latent factor. Our hypothesized model is presented in **Figure 2** below. In this model, we further included *a priori* assumptions that the errors between the three items dealing with child sacrifice would be correlated. We made this assumption based on our observations from our first study where these three items were repeatedly associated with one another but would not come out as a full independent factor of their own either for technical reasons (Eigenvalues <1) or for substantial reasons (being associated with the Footbridge dilemma).

### Method

#### Participants and Procedure

All participants were recruited from the e-mailing lists of student unions in Finland. Three hundred and forty-six people (*N* = 346; 54 male; *M*_age_ = 25.23; *SD*_age_ = 5.85; *range* = 18 – 65) successfully completed a correlational study prepared with the commercial questionnaire software Qualtrics. After giving their informed consent, participants filled in some exploratory measures not related to the present study (i.e., perception of time and childhood stability), after which they progressed to the high-conflict dilemmas. Participants had a chance to win one out of five movie tickets in a raffle as compensation.

#### Materials

##### Moral preference measure

We used the same materials in Study 2 as we did previously. For a full description of the materials see Pilot Study and Supplementary Material. Cronbach’s α for the items in this sample was 0.89 and Tarkkonen’s ρ = 0.83, both indicating acceptable internal consistency.

### Confirmatory Factor Analysis and Its Evaluation Criteria

For our CFA, we used the statistical programming language R and a peer-reviewed structural equation modeling library called *lavaan* (Rosseel, 2012). Lavaan is a reliable Open Source alternative for Mplus and provides the same model evaluation criteria. Here, we report the most common ones recommended by Kline (2010) which are: (1) *X*^2^; (2) The comparative fit index (CFI); (3) The root mean square error of approximation (RMSEA); and (4) Standardized root mean square residual (SRMR). We also report TLI as recommended by Byrne (2012).

*X*^2^ is traditionally used in CFA as a fit index and it is expected to be as close to zero as possible and thus not expected to be significant (i.e., *p*-value should be >0.05); however, in practice with sample sizes >200 it is almost always statistically significant. Nonetheless *X*^2^ can still be helpful in estimating fits between several models. CFI is an index that gets values from 0 to 1 measuring discrepancy between the hypothesized model and the actual data. CFI is not influenced by the sample size, 0.90 is usually considered to be a passable value, however, values above 0.95 are commonly expected in peer review. RMSEA is an absolute measure of a model fit, which improves as the number of variables in the model or the number of observations in the sample go up. Cut-off points of 0.01, 0.05, and 0.08 have been suggested, corresponding to excellent, good, and mediocre fits, respectively (MacCallum et al., 1996); confidence intervals should be used to understand the size of sampling error (upper-bound should preferable be <0.1). The SRMR indicates the difference between observed and predicted values; zero indicating perfect fit; values <0.08 are considered to indicate a good fit (Hu and Bentler, 1999). TLI, or Tucker-Lewis Index is a similar measure to CFI but it imposes heavier penalties for complex models; 0.95 is considered to be the cut-off point for indicating good fit (Hu and Bentler, 1999).

### Results

**FIGURE 3:**
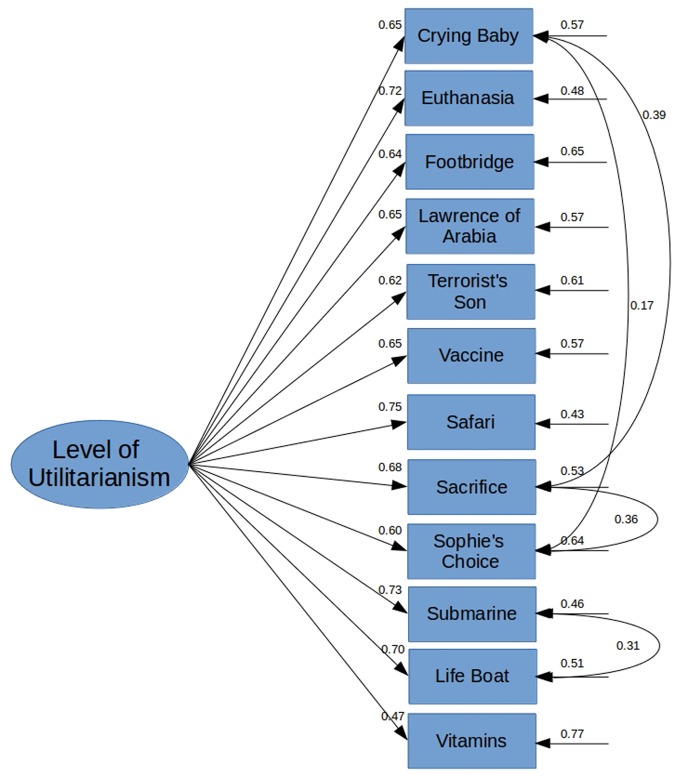
**Results for Study 2**.

We ran a CFA on the hypothesized model presented in **Figure 2** with a robust Maximum Likelihood (MLM) estimation. This hypothesized initial model did have a satisfactory fit with the hypothesized model (*X^2^*(51) = 123.38, CFI = 0.96, TLI = 0.95, RMSEA = 0.06, 90% CI [0.05 – 0.08], SRMR = 0.04). We then proceed to investigate potential model modifications. We decided to add error covariance between the Life Boat and Submarine dilemmas into the model, since both deal with an emergency situation at the sea (Suggested MI: 27.67). This increased the model fit statistically significantly (Δ*X^2^* = 19.86, *p* < 0.001; *X^2^*(50) = 100.90, CFI = 0.97, TLI = 0.96, RMSEA = 0.05, 90% CI [0.04 – 0.07], SRMR = 0.036). All the estimates of the model were statistically significant (for standardized estimates/factor loadings see **Figure 3** below).

### Discussion of the Results for Study 2

The results of Study 2 imply that there is a unitary single-factor structure behind the items measuring utilitarianism in high-conflict moral dilemmas. Furthermore, the hypothesized error covariances were all statistically significant. This indicates that although the three items that deal with child sacrifice do not cluster together strongly enough to warrant separating them into their own factor, they are nonetheless associated. This could be an indicator that the general machinery responsible for utilitarian-deontological judgments might take in situational cues which change the weights in the way these judgments are reached or made (e.g., Kurzban et al., 2012).

Our further modification of the measurement model, adding the covariance term between the Submarine and Life Boat dilemmas, was also substantially and statistically warranted. The factor loadings of the measurement model are moderately strong and relatively similar to those found in out laboratory data (see **Figure 3**). However, since this model was modified and error covariance terms were added to the model, we wanted to replicate the results of our CFA with another data set to rule out the possibility of over fitting our model.

## Study 3

The results of the two previous studies suggest that there is a uniform general structure behind the sacrificial moral dilemmas and that the three items dealing with child sacrifice are associated. Furthermore, a shared error covariance term between two sea-related dilemmas was added to the model in Study 2. We ran a third study to confirm the model constructed in Study 2 in order to rule out possible idiosyncrasies or overfitting that could possibly account for the results of Study 2. Finally, we also tested for the equality of the factor loadings between Studies 2 and 3.

### Method

#### Participants and Design

The data was collected from The Netherlands (*N* = 174) and from Finland (*N* = 343). All Dutch participants were recruited through the social media. All Finnish participants were recruited from the e-mailing lists of student unions in Finland. To sum up, five hundred and seventeen people in total (*N* = 517; 180 male; *M*_age_ = 26.30; *SD*_age_ = 8.42; *range* = 18 – 66) successfully completed a study prepared with the commercial questionnaire software Qualtrics. The data was collected in conjunction with larger on-line experiment, results of which will be reported elsewhere. After giving their informed consent, participants filled in some exploratory measures (i.e., rating content of pictures and other emotional sensitivity measures) not related to the present study, after which they progressed to the sacrificial dilemmas. All participants responded to all the questions in their native language. Participants had a chance to win one out of three movie tickets in a raffle as compensation.

#### Materials

##### Moral preference measure

We used the same materials as in the previous studies. For a full Description of the materials see Pilot Study and Supplementary Material. Cronbach’s α for the items in this sample was 0.88 and Tarkkonen’s ρ = 0.79, both indicating acceptable internal consistency.

### Results

#### Testing the *A priori* Model from Study 2

We ran a CFA on the final model of Study 2 (**Figure 3**), with a robust Maximum Likelihood (MLM) estimation. This initial model did have a satisfactory fit with the model (*X^2^*(50) = 135.99, CFI = 0.96, TLI = 0.94, RMSEA = 0.06, 90% CI [0.05 – 0.07], SRMR = 0.04), hence, we did not proceeded with further model modification. These results indicate that the single-factor solution that was found in the previous study was also valid in this data. We then proceeded to test whether the factor loadings between the two data sets were equal.

#### Testing for Configural and Weak Invariance between Study 2 and Study 3

We combined the data sets from Study 2 and Study 3 and ran a CFA for two groups to test the fit of our configural model. Since the results indicated that the configural model fits the data acceptably (*X^2^*(100) = 236.85, CFI = 0.96, TLI = 0.95, RMSEA = 0.05, 90% CI [0.04 – 0.06], SRMR = 0.03), we proceeded to test the equality of the factor loadings across the two models (i.e., weak invariance). The results indicate that a CFA model, where loadings are constrained equal across groups, fits the data well (*X^2^*(111) = 261.21, CFI = 0.96, TLI = 0.95, RMSEA = 0.05, 90% CI [0.04 – 0.06], SRMR = 0.04). Cheung and Rensvold (2002) recommend using change in CFI for testing invariance across groups. This is due to the fact that Δ*X^2^* is effected by sample size whereas ΔCFI is not (our combined sample for Studies 2 and 3 is very large, *N* > 850). Changes in CFI < 0.01, as observed here, are considered trivial. In conclusion, there are no meaningful differences in factor loadings between Studies 2 and 3. See full listing of estimations and factor loadings in **Table 4**.

**Table 4:** Factor scores from standardized and non-standardized factor loadings of invariance testing from combined data sets of Studies 2 and 3.

					Standardized loadings	
					
Items	Estimate	S.E.	*Z*-value	*p*-value	Data 2	Data 3
Crying baby	1.00				0.64	0.58
Euthanasia	1.12	0.05	18.81	<0.001	0.70	0.66
Foot bridge	0.78	0.06	15.17	<0.001	0.59	0.55
Lawrence of Arabia	1.08	0.06	15.99	<0.001	0.69	0.64
Terrorist’s son	0.92	0.06	14.94	<0.001	0.59	0.50
Life boat	1.17	0.06	17.19	<0.001	0.70	0.66
Safari	1.23	0.04	18.84	<0.001	0.75	0.72
Sacrifice	0.95	0.05	19.58	<0.001	0.64	0.54
Sophie’s choice	0.97	0.06	17.33	<0.001	0.61	0.59
Submarine	1.13	0.06	16.93	<0.001	0.74	0.74
Vaccine	1.16	0.06	17.41	<0.001	0.68	0.65
Vitamins	0.81	0.06	12.74	<0.001	0.48	0.46


*Data 2 refers to the data set described in Study 2 and Data 3 refers to the data set described in Study 3.*

### Discussion of Results for Study 3

The results of Study 3 support the one factor solution suggested by Study 1 and Study 2. Furthermore, our results indicate that the three dilemmas associated with child sacrifice should be given consideration when using these items as a scale in experimental studies. We also ran a cross-validation analysis for the model found in Study 2, which essentially shows that the factor loadings are stable across samples and replicable with decent accuracy.

## General Discussion

In three studies (and a pilot), we examined the factorial structure of the 12 high-conflict moral dilemmas presented by Greene et al. (2008), by asking participants to denote their level of acceptance of a deontological violation on a Likert scale. The purpose was to examine the factorial structure of these responses to see whether all of the dilemmas truly measure the same thing. Across all three studies, we found support for a unidimensional factorial solution by using both (multiple) exploratory and confirmatory factor analytic techniques, as well as by using an alternative reliability criterion (Tarkkonen’s ρ) to Cronbach’s α. The results suggest that there might be a uniform cognitive mechanism involved and that none of the items should be dropped from the scale or the measurement model.

We found that all of the three dilemmas dealing with child sacrifice were associated with each other in all three of our studies, but not strongly enough to form their own sub-factor (supporting the kin-selection moderation hypothesis suggested by Kurzban et al., 2012). We also found that two of the dilemmas set in a maritime context had a repeatedly associated residual covariance (Studies 2 and 3). We ran a cross-validation/invariance analysis for the factorial loadings of the items that were measuring the latent factor of utilitarian moral preference, and showed that the results can be reliably replicated (along with controls for residual covariances). Future studies using these dilemmas as dependent measures should take the error covariances into consideration when building measurement scales. Also, serious consideration should be given to the strategy of using a weighted average of the dilemmas rather than a simple average, since not all items load on the latent factor equally. We suggest the unstandardized factor loadings from **Table 4** as possible candidates for weights.

At the time of writing, the field of moral psychology is progressing rapidly. Although the new revolution in moral psychology began from neuroscience, studies using these high-conflict moral dilemmas, or their variations, as dependent measures are being published in new journals and in new contexts with an increasing pace. Furthermore, utilitarianism is now being correlated with different individual trait measures and used in increasingly varied contexts. Thus, a psychometrically validated and tested instrument should be developed so that the results coming from different papers and from different fields can be reliably compared and assessed in relation to one another. Given that some changes in moral judgment have been linked with psychopathologies (e.g., Koenigs et al., 2011), neural lesions (e.g., Greene, 2007b; Koenigs et al., 2007), psychopharmacological agents (e.g., Perkins et al., 2012; Terbeck et al., 2013), and personality dimensions (e.g., Bartels and Pizarro, 2011), the research has become influential enough to have real life implications and, possibly, even policy recommendations. Therefore, it is not irrelevant which instruments are used and how moral preferences or judgments for utilitarian or deontological morality are operationalized.

Measurement of utilitarian/deontological preferences with a unidimensional instrument seems warranted, given certain provisions. Given that there are several unvalidated instruments in use, results from different past studies should be interpreted with caution. Caution should also be exerted in interpreting those past studies that have used the original set of high-conflict dilemmas provided by Greene et al. (2008). Previously theses items have either been dichotomous measures, or Likert scales that have been summed or averaged together, and the problems with the three child sacrificial dilemmas have not extensively discussed (see Christensen and Gomila, 2012 for a review). As far as we are aware, the psychometrical properties of these dilemmas have not been properly tested with IRT models (for dichotomous versions of the dilemmas) or with extensive factor analytical techniques (for continuous Likert scale versions), prior to our studies presented here. Our results provide some evidence that not all dilemmas load on to the latent factor equally, and therefore previous studies might have an error component large enough to influence previous statistical significance tests.

As with any study, this study suffers from the standard limitations of laboratory and Internet-based questionnaire studies. Respondents to these dilemmas are not a random sample from the general population: most likely participants in these studies are more curious, more patient and younger than the population average. Furthermore, questionnaire studies, where self-report measures are used, can suffer from a variety of demand characteristics and positive response biases. Notwithstanding, we took all the precautionary measures to minimize this by underlining the anonymity of the questionnaire and by telling the participants that, we are not screening individuals or interested in clinical profiling.

Our findings imply that there is some general cognitive structure behind responses to moral dilemmas, and none of our analyses technically supported the exclusion of any of the 12 dilemmas used, however, there might be reasons to separate the child sacrifice dilemmas from the other nine, or give them some special attention. Nevertheless, the exact nature of the general moral cognitive structure is beyond the scope of this paper.

## Conclusion

In sum, we successfully showed in three studies (plus a pilot) and in a cross validation analysis that there is a unitary general factor of utilitarian/deontological preference that combines all the dilemmas under the same latent construct. We further demonstrated that the factorial loadings are stable between studies, and suggested that the unstandardized estimates for the factor loadings could be used as weights for the dilemmas when averaged together in future empirical studies.

## Author Contributions

ML collected most of the data, analyzed it and wrote up the first draft of the paper. JS collected some of the data, double checked the analyses and complemented the manuscript.

## Conflict of Interest Statement

The authors declare that the research was conducted in the absence of any commercial or financial relationships that could be construed as a potential conflict of interest.
