# Supplementary material for: Are Utilitarian/Deontological Preferences Unidimensional?
Source: Front Psychol. 2016 Aug 17;7:1228. doi: 10.3389/fpsyg.2016.01228 (PMC4987326; doi:10.3389/fpsyg.2016.01228)
Supplement: Supplementary file 1 [file Presentation_1.PDF]

Crying Baby

Enemy soldiers have taken over your village. They have orders to kill all remaining civilians. You and some of your townspeople have sought refuge in the cellar of a large house. Outside you hear the voices of soldiers who have come to search the house for valuables.

Your baby begins to cry loudly. You cover his mouth to block the sound. If you remove your hand from his mouth his crying will summon the attention of the soldiers who will kill you, your child, and the others hiding out in the cellar. To save yourself and the others you must smother your child to death.

How acceptable is it for you to smother your child in order to save yourself and the other townspeople?

Euthanasia

You are the leader of a small group of soldiers. You are on your way back from a completed mission deep in enemy territory when one of your men has stepped in trap that has been set by the enemy and is badly injured. The trap is connected to a radio device that by now has alerted the enemy to your presence. They will soon be on their way.

If the enemy finds your injured man they will torture him and kill him. He begs you not to leave him behind, but if you try to take him with you your entire group will be captured. The only way to prevent this injured soldier from being tortured is to shoot him yourself.

How acceptable is it for you to shoot this soldier in order to prevent him from being tortured by the enemy?

Footbridge

A runaway trolley is heading down the tracks toward five workmen who will be killed if the trolley proceeds on its present course. You are on a footbridge over the tracks, in between the

approaching trolley and the five workmen. Next to you on this footbridge is a stranger who happens to be very large.

The only way to save the lives of the five workmen is to push this stranger off the bridge and onto the tracks below where his large body will stop the trolley. The stranger will die if you do this, but the five workmen will be saved.

How acceptable is it for you to push the stranger on to the tracks in order to save the five workmen?

Lawrence of Arabia

You are the leader of a small army that consists of warriors from two tribes, the hill tribe and the river tribe. You belong to neither tribe. During the night a hill tribesman got into an argument with a river tribesman and murdered him. The river tribe will attack the hill tribe unless the murderer is put to death, but the hill tribe refuses to kill one of its own warriors.

The only way for you to avoid a war between the two tribes that will costs hundreds of lives is to publicly execute the murderer by cutting off is head with your sword.

How acceptable is it for you to cut off this man’s head in order to prevent the two tribes from fighting a war that will cost hundreds of lives?

Terrorist's Son

You are negotiating with a powerful and determined terrorist who is about to set off a bomb in a crowded area. Your one advantage is that you have his teen-age son in your custody.

There is only one thing that you can do to stop him from detonating his bomb, which will kill thousands of people if detonated. To stop him, you must contact him over the satellite hook-up that he has established and, in front of the camera, break one of his son’s arms and then threaten to break the other one if he does not give himself up.

How acceptable is it for you to break the terrorist’s son’s arm in order to prevent the terrorist from killing thousands of people with his bomb?

Lifeboat

You are on a cruise ship when there is a fire on board, and the ship has to be abandoned. The lifeboats are carrying many more people than they were designed to carry. The lifeboat you’re in is sitting dangerously low in the water—a few inches lower and it will sink.

The seas start to get rough, and the boat begins to fill with water. If nothing is done it will sink before the rescue boats arrive and everyone on board will die. However, there is an injured person who will not survive in any case. If you throw that person overboard the boat will stay afloat and the remaining passengers will be saved.

How acceptable is it for you to throw this person overboard in order to save the lives of the remaining passengers?

Safari

You are part of a group of ecologists who live in a remote stretch of jungle. The entire group, which includes eight children, has been taken hostage by a group of paramilitary terrorists. One of the

terrorists takes a liking to you. He informs you that his leader intends to kill you and the rest of the hostages the following morning.

He is willing to help you and the children escape, but as an act of good faith he wants you to kill one of your fellow hostages whom he does not like. If you refuse his offer all the hostages including the children and yourself will die. If you accept his offer then the others will die in the morning but you and the eight children will escape.

How acceptable is it for you to kill one of your fellow hostages in order to escape from the terrorists and save the lives of the eight children?

Sacrifice

You, your husband, and your four children are crossing a mountain range on your return journey to your homeland. You have inadvertently set up camp on a local clan's sacred burial ground.

The leader of the clan says that according to the local laws, you and your family must be put to death. However, he will let yourself, your husband, and your three other children live if you yourself will kill your oldest son.

How acceptable is it for you to kill your oldest son in order to save your husband and your other three children?

Sophie's Choice

It is wartime and you and your two children, ages eight and five, are living in a territory that has been occupied by the enemy. At the enemy's headquarters is a doctor who performs painful experiments on humans that inevitably lead to death.

He intends to perform experiments on one of your children, but he will allow you to choose which of your children will be experimented upon. You have twenty-four hours to bring one of your children to his laboratory. If you refuse to bring one of your children to his laboratory he will find

them both and experiment on both of them.

How acceptable is it for you to bring one of your children to the laboratory in order to avoid having them both die?

Submarine

You are the captain of a military submarine traveling underneath a large iceberg. An onboard explosion has caused you to lose most of your oxygen supply and has injured one of your crew who is quickly losing blood. The injured crew member is going to die from his wounds no matter what happens.

The remaining oxygen is not sufficient for the entire crew to make it to the surface. The only way to save the other crew members is to shoot dead the injured crew member so that there will be just enough oxygen for the rest of the crew to survive.

How acceptable is it for you to kill the fatally injured crew member in order to save the lives of the remaining crew members?

Vaccine

A viral epidemic has spread across the globe killing millions of people. You have developed two substances in your home laboratory. You know that one of them is a vaccine, but you don't know which one. You also know that the other one is deadly.

Once you figure out which substance is the vaccine you can use it to save millions of lives. You have with you two people who are under your care, and the only way to identify the vaccine is to inject each of these people with one of the two substances. One person will live, the other will die, and you will be able to start saving lives with your vaccine.

How acceptable is it for you to kill one of these people with a deadly injection in order to identify a vaccine that will save millions of lives?

Vitamins

You are the leader of a mountaineering expedition that is stranded in the wilderness. Your expedition includes a family of six that has a genetically caused vitamin deficiency. A few people's kidneys contain large amounts of this vitamin.

There is one such person in your party. The only way to save the lives of the six members of this family is to remove one of this man's kidneys so that the necessary vitamins may be extracted from it. The man will not die if you do this, but his health will be compromised. The man is opposed to this plan, but you have the power to do as you see fit.

How acceptable is it for you to forcibly remove this man's kidney in order to save the lives of the six vitamin-deficient people?
